# Meta-analysis and network pharmacology studies of the clinical efficacy of Guizhi Fuling capsules/pills combined with dienogest in treating endometriosis

**DOI:** 10.1097/MD.0000000000040528

**Published:** 2024-12-06

**Authors:** Yajie Qin, Xiaotian Yang, Qi Zhao, Xingran Tang, Huijin Zhao, Yang Jiao, Huifang Zhou

**Affiliations:** a The First Clinical Medical College, Nanjing University of Chinese Medicine, Nanjing, Jiangsu, China.

**Keywords:** dienogest, endometriosis, Guizhi Fuling capsules/pills, meta-analysis, network pharmacology analysis

## Abstract

**Background:**

Endometriosis (EMs) is a common chronic inflammatory gynecological disease that belongs to the classification of Traditional Chinese Medicine Syndromes “*Zheng Jia*,” and the classic Chinese formula Guizhi Fuling (GZFL) demonstrates significant clinical efficacy in the treatment of this condition. This study aims to investigate GZFL’s effect and potential mechanism in EMs.

**Methods:**

The search reviewed randomized controlled trials in 7 databases from inception to 2024, assessed quality with the Cochrane tool, and analyzed data with STATA 15 by 2 reviewers. In the network pharmacology study, we searched and screened the components and targets of GZFL, subsequently compared these targets to EMs targets, and used bioinformatics techniques to analyze and explore their potential interactions.

**Results:**

Nine randomized controlled trials involving 897 participants were analyzed. Meta-analysis showed that GZFL combined with dienogest significantly enhanced the clinical effectiveness rate (odds ratio = 2.404, 95% confidence intervals [CI], 1.868 to 3.093; *P <* .001). Specifically, combination therapy with GZFL reduced serum carbohydrate antigen 125 (standardized mean differences [SMD] = −1.65, 95% CI = −2.13 to −1.17, *P <* .001), estradiol (SMD = −1.54, 95% CI = −1.89 to −1.19, *P = *.003), matrix metalloproteinases (SMD = −2.636, 95% CI = −2.993 to −2.279, *P <* .001), pain scores (SMD = −0.88, 95% CI = −1.11 to −0.67, *P <* .001) and the diameter of ectopic cysts (SMD = −1.7, 95% CI = −2.42 to −0.98, *P <* .001). Network pharmacology analysis identified 136 components and 145 common targets, focusing on interleukin-6, cellular tumor antigen p53, epidermal growth factor receptor, estrogen receptor alpha, Cyclooxygenase-2, and matrix metalloproteinases-9. Gene ontology and Kyoto Encyclopedia of Genes and Genomes analyses suggested GZFL modulates hormone receptors and inflammatory responses in EMs treatment.

**Conclusion:**

In conclusion, GZFL combination treatment could increase the clinical effectiveness rate of EMs patients, and reduce the serum level of carbohydrate antigen 125, estradiol, matrix metalloproteinases, pain scores, and the diameter of the ectopic cyst. The potential mechanism might be linked to the modulation of hormone receptors and inflammation.

## 1. Introduction

Endometriosis (EMs) is a complicated clinical syndrome marked by the presence of endometrial-like tissue, typically found within the uterine cavity, aberrantly implanted outside its natural location, including the ovaries, fallopian tubes, and pelvic cavity.^[1]^ Abnormal endometrial tissue growth due to hormonal changes during the menstrual cycle causes inflammation, pain, and adhesion,^[2]^ resulting in a wide range of clinical manifestations, such as pelvic pain, painful menstruation, dyspareunia, and infertility.^[3]^

As a prevalent gynecological condition, EMs affects over 5% to 10% of women of reproductive age globally.^[4]^ However, owing to the overlap with other gynecological conditions and nonspecific symptoms, the diagnosis of this condition is often delayed until the late reproductive years.^[5]^ Thus, it significantly affects women’s ability to work and their overall quality of life.^[6]^

There are several treatments for EMs, including NAIDS, hormone therapy, and surgery.^[7]^ As a progestogenic drug, dienogest (DNG) is widely used for the management of EMs.^[8]^ However, there is limited evidence that hinders treatment recommendations.^[9]^ Hormone therapy may lead to significant adverse effects stemming from its interference with estrogen levels. Furthermore, a high recurrence rate after discontinuation of the drug should be noted. Guizhi Fuling pills/capsules (GZFL) is a traditional Chinese medicine formulation recommended for the management of EMs, and for adjuvant treatment of endosis after surgery.^[10]^ A randomized controlled trial (RCT) that included 353 dysmenorrhea patients showed that GZFL substantially mitigated the intensity of dysmenorrhea.^[11]^ Additionally, studies have shown that GZFL can improve endometrial hyperplasia^[12]^ and reduce the serum levels of PGF2α and COX-2 in dysmenorrhea rats.^[13]^

GZFL contains multiple components, targets, and effects. Thus, it is essential to explore the potential mechanisms and therapeutic efficacy of GZFL to improve the diagnosis and treatment of EMs. However, there is a lack of clinical evidence from evidence-based medical studies assessing the efficacy of GZFL in treating EMs. Therefore, we performed a systematic meta-analysis of published studies to investigate the effectiveness of GZFL supplementation in EM patients. We also explored the potential genes and mechanisms of action of GZFL in treating EMs via network pharmacology.

## 2. Methods

### 2.1. Meta-analysis

#### 2.1.1. Search strategy

This study adhered to the rigorous guidelines outlined in the Preferred Reporting Items for Systematic Reviews and Meta-Analyses (PRISMA) 2020.^[14]^ Additionally, this meta-analysis was registered on PROSPERO, the database of The Center for Reviews and Dissemination (No. CRD42024530992). We searched 7 databases: PubMed, Web of Science, Embase, Cochrane Library, China National Knowledge Infrastructure, Wanfang, and Chinese Scientific Journals Database. The search encompassed a blend of database-specific controlled vocabulary terms and free-text keywords about EMs (e.g., “Endometrioses,” “Endometrioma,” and “Chocolate cyst of ovary”) and Guizhi filing (e.g., “Guizhi fuling pills,” “Guizhi fuling capsules,” and “Dienogest”). Details of the search strategy are presented in Supplementary Material S1, Supplemental Digital Content, http://links.lww.com/MD/O16. The retrieval period for each database spanned from its inception to April 24, 2024, and no limitations were placed on language or publication status. All procedures included in the study involving human participants followed the ethical standards of the institutional and national research committees.

#### 2.1.2. Criteria of inclusion and exclusion

Inclusion criteria: (1) RCTs and (2) eligible studies that included participants diagnosed with EMs according to the Guidelines for the Diagnosis and Treatment of EMs (2015)^[15]^ or imaging examinations. (3) The control group was treated with DNG at a dose recommended in the Guidelines for the Management of EMs for pain,^[10]^ while the intervention group was administered a combination of GZFL. The same DNG dosage as the control group and the dosage of GZFL were not restricted, regardless of the scheduled time for administering and mode of administration. (4) Studies that included at least one of the following outcomes: clinical efficiency rate (CER), carbohydrate antigen 125 (CA125), estradiol (E2), pain scores, matrix metalloproteinase (MMP), and diameter of the ectopic cyst.

Exclusion criteria: (1) commentaries, reviews, and in vitro and animal studies; (2) Duplicate publications; (3) incomplete data-based research studies; and (4) Intervention.

#### 2.1.3. Data extraction

##### 2.1.3.1. Study selection

All articles were imported into Endnote X9.1 for filtering. Two reviewers (Y.Q. and X.Y.) first removed the duplicate studies. Next, by browsing the titles and abstracts, conferences, reviews, animal experiments, and related empirical articles were excluded from the study. In the third round of screening, articles irrelevant to the topic of research content and methodology were eliminated after a thorough reading of the full text.

##### 2.1.3.2. Data extraction

Data from the included studies were compiled into a standardized form, outlining the first author’s name, publication date, study design, participant age (treatment/control; years), inventions, treatment duration, and outcomes. All data were independently extracted by 2 authors (Y.Q.and X.Y.) and further reviewed by a third author (Q.Z.).

#### 2.1.4. Risk of bias assessment

The included studies were assessed for bias according to the methods used in the Cochrane Handbook for Systematic Reviews of Interventions version 6.4^[16]^ of RCTs for quality assessment, including whether random allocation methods were used, allocation protocols were concealed, blinding methods were used, outcome data were complete, study results were selectively reported, and other sources of bias were considered. The bias assessment was completed by Review Manager 5.4, and disagreements were discussed with X.Y.

#### 2.1.5. Quality assessment

Quality assessment was performed utilizing the GRADE criteria^[17,18]^ according to the website (https://www.gradepro.org/).

#### 2.1.6. Data synthesis and meta-analysis

For ordered data, odds ratios and 95% confidence intervals (CIs) were used to represent effect sizes, whereas standardized mean differences (SMDs) and 95% CIs were used for continuous data. The fixed-effect model was used for the effect model when insignificant heterogeneity was <50%(I-square [I^2^] < 50%). If I^2^ were >50% (I^2^ > 50%), meta-regression would be performed according to the possible factors derived from the sensitivity analysis. If *P* > .05, a random-effects model was employed. If *P <* .05, further subgroup analysis will be conducted using a random-effects model. Data synthesis and meta-analysis were conducted using STATA 15.

### 2.2. Network pharmacology

#### 2.2.1. Gene search of EMs

The genes associated with EMs were searched by inputting the keyword “Endometrioses,” “Endometrioma,” and “Chocolate cyst of ovary” in 2 online databases: Mendelian Inheritance in Man (OMIM, https://omim.org/) and GeneCards (https://www.genecards.org/).

#### 2.2.2. Identify the active ingredients and potential targets of GZFL

In the Traditional Chinese Medicine Systems Pharmacology (TCMSP, https://old.tcmsp-e.com/tcmsp.php) database, the effective components of 5 constituent drugs of GZFL (*Cinnamomum cassia* Presl [Lauraceae; CINNAMOMI RAMULUS], *Poria cocos* (Schw.) Wolf [Polyporaceae; PORIA], *Paeonia suffruticosa* Andr. [Ranunculaceae; MOUTAN CORTEX], *Paeonia lactiflora* Pall. [Ranunculaceae; PAEONIAE RADIX RUBRA], *Prunus persica* (L.) Batsch [Rosaceae; PERSICAE SEMEN]) were screened out, by establishing the criteria of oral bioavailability ≥ 30% and drug likeness ≥ 0.18.^[19]^ The canonical SMILES of these active components were collected from the PubChem database (https://pubchem.ncbi.nlm.nih.gov/) and entered into Swiss Target Prediction (http://www.swisstargetprediction.ch) to predict possible targets. For a more comprehensive search, the BAT-man database (http://bionet.ncpsb.org.cn/batman-tcm/index.php) was used to determine the composition and corresponding targets of GZFL according to a score ≥ 20. The predicted potential targets were intersected with EMs disease genes to identify the potential effective targets of GZFL. Cytoscape (version 3.9.1) was used to visualize the ingredient-target network.

#### 2.2.3. Construct the protein–protein interaction (PPI) network and identify key targets

All the targets with an interaction score > 0.4 were screened out via the STRING database (http://cn.string-db.org). PPI network using Cytoscape 3.9.1 software. Network topology parameters of targets, including degree (DC), betweenness centrality, closeness centrality (CC), and eigenvector centrality, were analyzed using the CytoNCA algorithm in the Cytoscape software. Thus, important nodes in the network were identified.

#### 2.2.4. Gene ontology (GO) and Kyoto Encyclopedia of Genes and Genomes (KEGG) analysis

The key targets, processed after 2.2.3, were imported into the Metascape database (https://metascape.org/) for GO and KEGG analysis. According to the terms with a *P* < .01 and an enrichment factor > 1.5, relevant cell component (CC), molecular function (MF), biological process (BP), and KEGG pathway data were obtained. Finally, the directories of the GO and KEGG enrichment analyses were selected, and bubble graphs were drawn using the bioinformatics mapping website (http://www.bioinformatics.com.cn/).

#### 2.2.5. Molecular docking simulation

Molecular docking was employed to theoretically simulate the interactions between potential bioactive components and crucial genes, thereby enabling the prediction of their binding patterns and affinities. Four important GZFL targets and their chemical components for the treatment of EMs in the PPI network were chosen for molecular docking. A structural file of the GZFL potential bioactive components was downloaded from the PubChem database. Using OpenBabelGUI software, the SDF format was converted to the PDB format. PDB files of the three-dimensional structures of the key proteins were retrieved from the Protein Database (RCSB) (http://www.rcsb.org/). The PDBIDs used were cellular tumor antigen p53 (TP53) (2H2D), estrogen receptor alpha (ESR1) (1X7R), Cyclooxygenase-2 (PTGS2) (3HS5), and MMP9 (5TH6). Utilizing AutoDockTools 1.5.6, polar hydrogens were added, and the grid box size was adjusted, resulting in the aforementioned PDB files being saved in pdbqt format. AutoDock Vina was used for molecular docking simulations and the minimum binding energy was calculated. Finally, the results were plotted with PyMOL.

## 3. Results

### 3.1. Meta-analysis

#### 3.1.1. Search results

**Figure 1.**
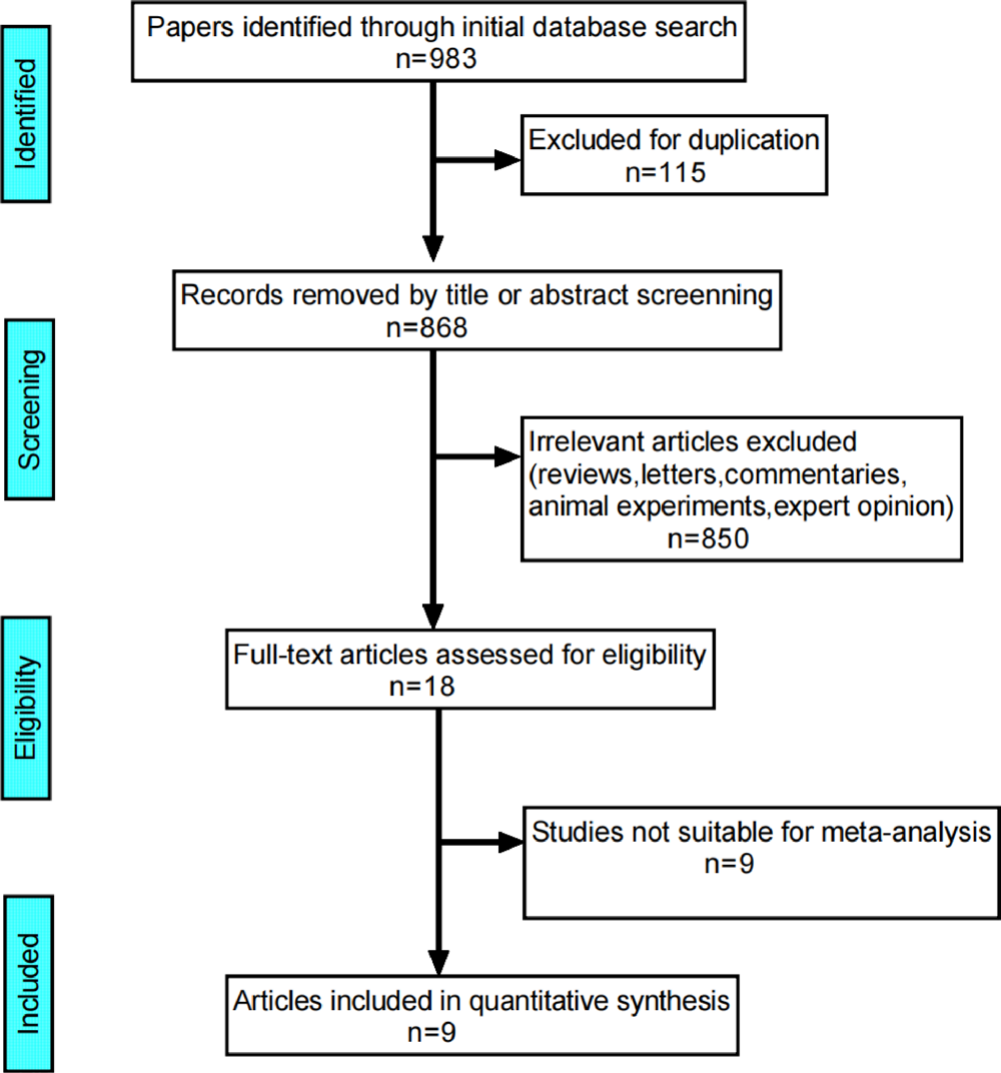
Flow diagram of the literature identification and selection.

Nine hundred eighty-three articles from 7 databases were imported into Endnote X9.1 for screening. After eliminating duplicates, the titles and abstracts of 868 articles were screened. Among them, 18 articles were recognized as potentially relevant and subsequently reviewed in full text. Finally, 9 studies with 897 participants (449 in the observation group and 448 in the control group) were included to explore the association between EMs and GZFL (Fig. 1).

#### 3.1.2. Characteristics of the selected studies

Detailed descriptions of the key characteristics of all the included studies are shown in Table 1. Briefly, the study involved participants aged 28 to 40 years old. The control group was treated with DNG, whereas the intervention group underwent DNG + GZFL therapy for 3 to 6 months.

**Table 1:** The characteristics of the included studies.

Study	Sample (T/C)	Study design	Age (T/C, years)	Interventions		Duration	Outcomes
				Treatment	Comparator		
Chen (2023)	69/69	RCT	28 ± 5/ 28 ± 5	GZFLP(9 pills/d,bid + DNG(2mg/d,qd)	DNG(2mg/d,qd)	3 months	1.2.3
Jin (2020)	47/47	RCT	35.69 ± 3.02/ 35.71 ± 3.14	GZFLC(0.93 g/d,tid + DNG(2mg/d,qd)	DNG(2mg/d,qd)	90 days	1.3
Lu (2020)	88/87	RCT	29.04 ± 6.31/ 28.93 ± 6.37	GZFLC(0.93 g/d,tid + DNG(2mg/d,qd)	DNG(2mg/d,qd)	3 months	1.2.3.4.6
Tong (2021)	50/50	RCT	28.5 ± 5.2/ 28.4 ± 5.4	GZFLC(0.93 g/d,tid + DNG(2mg/d,qd)	DNG(2mg/d,qd)	3 months	1.3.4
Wang (2021)	30/30	RCT	38.55 ± 3.43/ 38.62 ± 3.35	GZFLC(0.93 g/d,tid + DNG(2mg/d,qd)	DNG(2mg/d,qd)	Unclear	1.2.3.5
Cai (2022)	43/43	RCT	27.43 ± 1.76/ 27.87 ± 1.54	GZFLC(0.93 g/d,tid + DNG(2mg/d,qd)	DNG(2mg/d,qd)	3 months	1.3
Yan (2024)	34/34	RCT	35.47 ± 4.21/ 36.11 ± 4.89	GZFLC(0.93 g/d,tid + DNG(2mg/d,qd)	DNG(2mg/d,qd)	6 months	1.2.5
Yao (2022)	45/45	RCT	28.47 ± 5.23/ 28.03 ± 5.51	GZFLP(9 pills/d,bid + DNG(2mg/d,qd)	DNG(2mg/d,qd)	3 months	1.2.3.4.6
Zhao (2023)	43/43	RCT	31.34 ± 7.14/ 30.76 ± 8.01	GZFLC(0.93 g/d,tid + DNG(2mg/d,qd)	DNG(2mg/d,qd)	12 weeks	1.4.6

1. Clinical Efficiency Rate (CER); 2. Carbohydrate antigen 125 (CA125); 3. Estradiol (E2); 4. Pain scores; 5. Matrix metalloproteinases (MMP); 6. Diameter of ectopic cyst.

DNG = dienogest, RCTs = randomized controlled trials.

#### 3.1.3. Risk bias assessment

**Figure 2.**
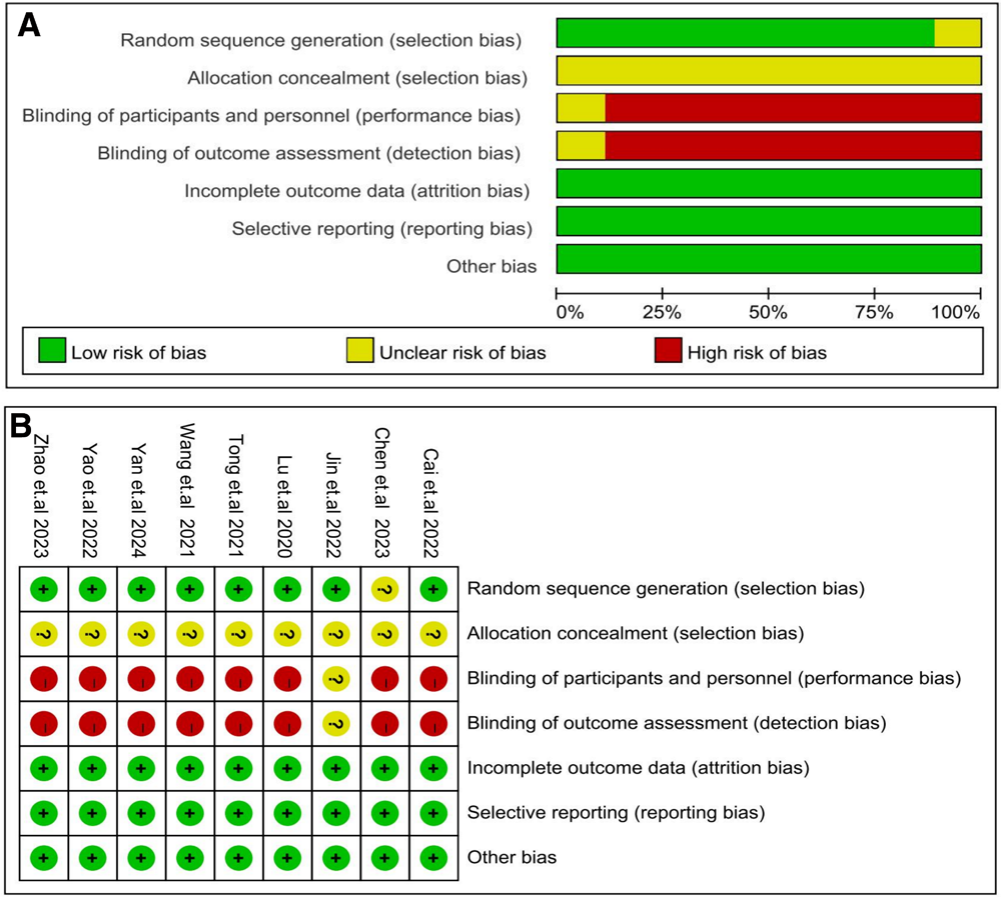
(A) Risk of bias of overall; (B) risk of bias graph of individual trials.

The risk of bias in all 9 included studies was assessed, as shown in Figure 2. All 9 studies included were RCTs. Seven studies^[20–26]^ had a clear method of randomization and were regarded as having a low risk of bias. Two studies^[27,28]^ reported an unclear method of randomization with a potentially low risk of bias. Owing to the different costs of clinical treatment, a blinding method was not well implemented in all studies, resulting in an unclear risk of bias. However, none of the studies selectively reported the results, and there were no other sources of bias.

#### 3.1.4. Outcome measures

**Figure 3.**
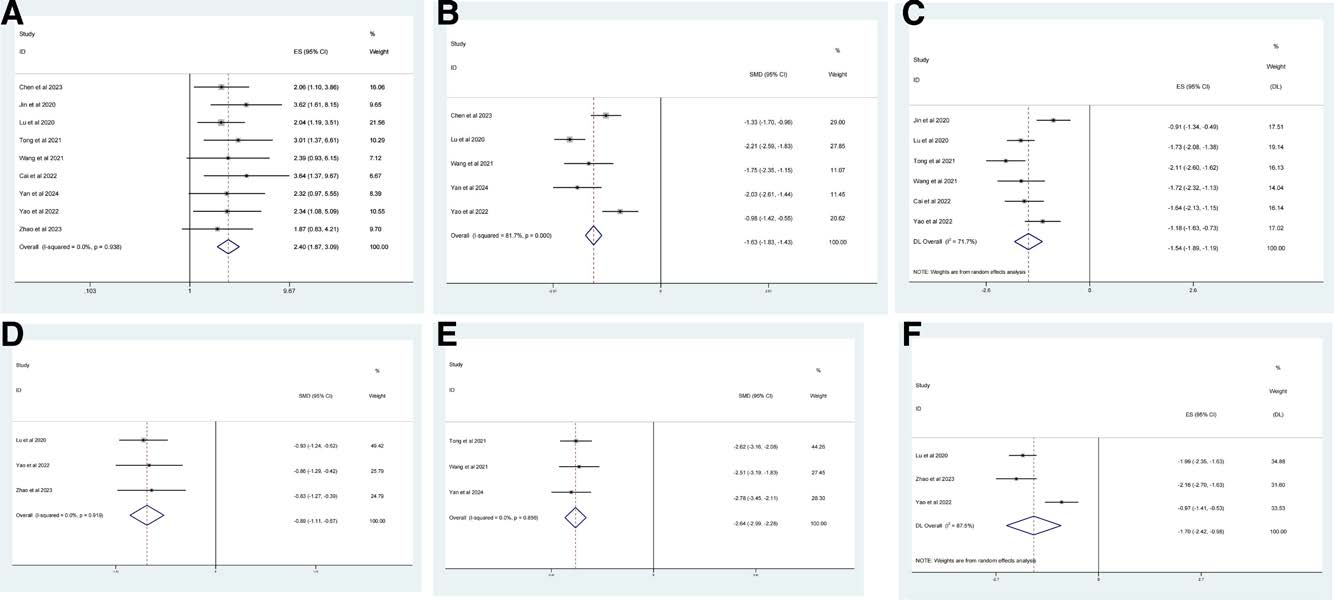
Forest plots for GZFL combination therapy versus DNG therapy alone. (A) CRT; (B) the CA125 level in serum; (C) the E2 level in serum; (D) the pain scores; (E) the MMP level in serum; and (F) the diameter of ectopic cyst. CA125 = carbohydrate antigen 125, DNG = dienogest, GZFL = Guizhi Fuling capsule/pills, MMP = matrix metalloproteinases.

CER 9 studies^[21–28]^ including 897 participants reported the clinical efficacy of GZFL in combination with EMs versus DNG alone. The heterogeneity test of 9 studies showed that I^2^ = 0% and *P* = .938; therefore, a fixed-effects model was used for the meta-analysis. Our analysis revealed that GZFL adjuvant therapy significantly increased the CER in women with EMs (odds ratio = 2.404, 95% CI, 1.868 to 3.093, *P <* .001) (Fig. 3A).

##### 3.1.4.1. Carbohydrate antigen 125

A meta-analysis of 531 participants in 5 studies^[21,24,25,27,28]^ reported that GZFL combination therapy significantly reduced the level of CA125 (SMD = −1.65, 95% CI = −2.13 to −1.17, *P <* .001). However, the high heterogeneity (I^2^ = 81.7%, *P <* .001) of these 5 studies should be noted. Subgroup analyses according to age and disease duration were performed before the meta-analysis. However, no significant disparities were observed in the meta-regression analyses (Supplementary Material S2, Supplemental Digital Content, http://links.lww.com/MD/O17), and a random-effects model was adopted for this analysis (Fig. 3B).

##### 3.1.4.2. Estradiol

Seven studies^[20–23,25,27,28]^ involving 743 participants reported differences in E_2_ levels between the control and GZFL intervention groups. After pooled analysis, I^2^ = 97.1%, *P <* .001, indicating the presence of strong heterogeneity among the selected studies. Sensitivity analyses (Supplementary Material S3, Supplemental Digital Content, http://links.lww.com/MD/O18) revealed lower heterogeneity after excluding the studies by Chen et al^[19]^ (I^2^ = 71.7%, *P* = .003). We reviewed the entire clinical study again and concluded that the main source of the reduction in heterogeneity may have been the baseline difference in the participants’ serum E_2_ levels. The level of E_2_ with combination therapy may be strongly correlated with disease severity. After exclusion,^[19]^ subsequent meta-analyses using a random-effects model revealed that combination therapy could significantly decrease E_2_ levels (SMD = −1.54, 95% CI = −1.89 to −1.19, *P* = .003) (Fig. 3C).

##### 3.1.4.3. Pain scores

These studies^[21,25,26]^evaluated the pain scores of 351 patients. Owing to the low heterogeneity (I^2^ = 0%, *P* = .919), a fixed-effects model was used in the meta-analysis. For the pain scores, the GZFL combination therapy group reported a greater reduction than the DNG alone treatment group (SMD = −0.88, 95% CI = −1.11 to −0.67, *P <* .001) (Fig. 3D).

##### 3.1.4.4. Matrix metalloproteinases

MMP was assessed in 3 studies^[22,24,28]^ including 228 participants. As shown in Figure 3E, a fixed-effect meta-analysis was used, based on I^2^ = 0% and *P* = .856. GZFL combination therapy had greater efficacy in reducing MMP (SMD = −2.636, 95% CI = −2.993 to −2.279, *P <* .001).

##### 3.1.4.5. Diameter of the ectopic cyst

The outcomes of the meta-analysis of 3 studies^[21,25,26]^ involving 381 participants showed that GZFL intervention significantly reduced the diameter of ectopic cysts (SMD = −1.703, 95% CI = −2.421 to −0.984, *P <* .001) (Fig. 3F). Considering the high degree of heterogeneity (I^2^ = 87.5%, *P <* .001), we attempted subgroup analyses by disease course or dosage after sensitivity analysis. However, a random effects model was ultimately used in the meta-analysis because the meta-regression analysis showed *P* = .157(Supplementary Material S2, Supplemental Digital Content, http://links.lww.com/MD/O17).

#### 3.1.5. Grade assessment and publication bias

**Figure 4.**
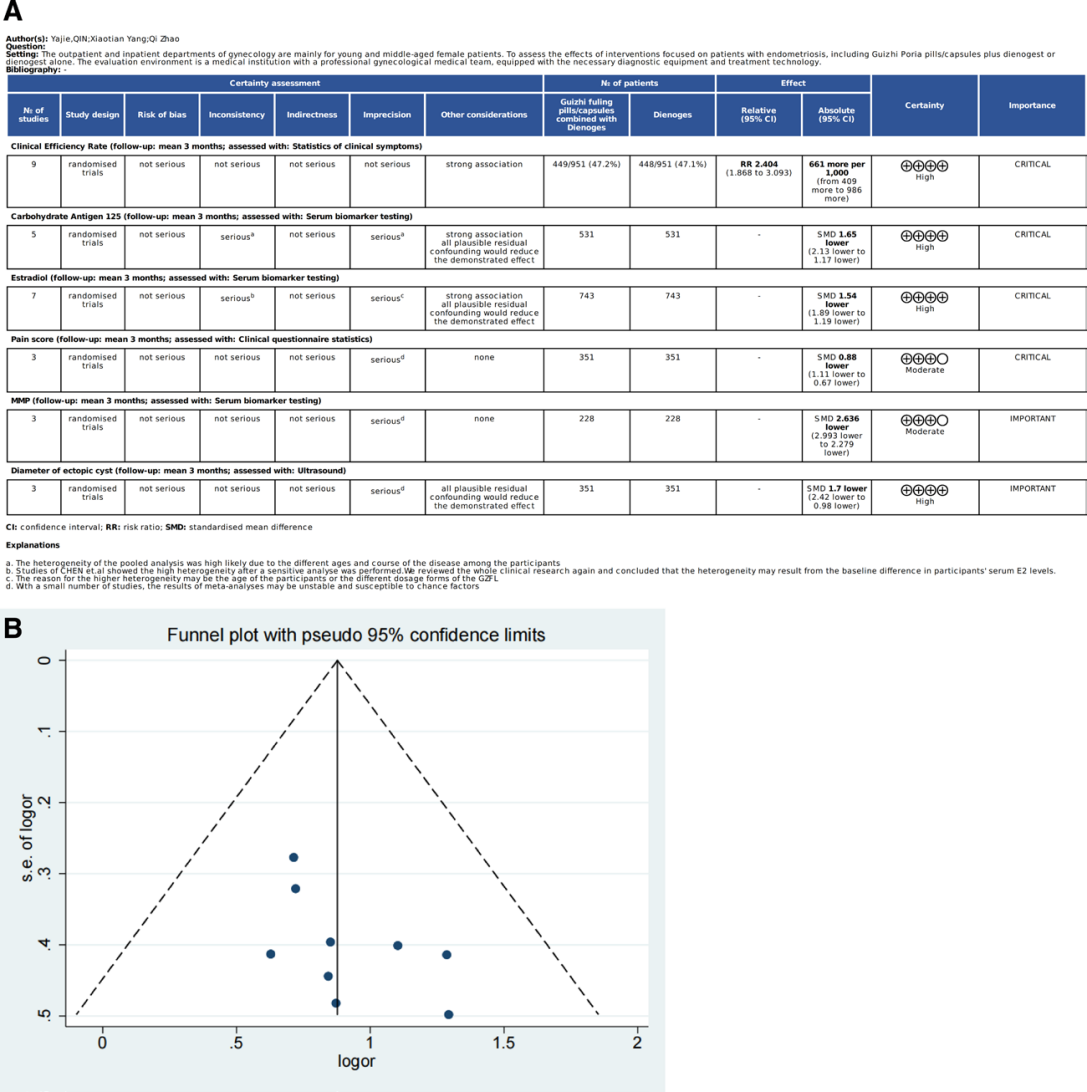
(A) Results of evidence quality evaluation of outcome indicators by GRADE. (B) Funnel plots of clinical effective rate.

The results of evidence quality evaluation of outcome indicators by GRADE ranged from “High” and “Moderate,” as shown in Figure 4A. The publication bias of studies that reported the CER was assessed by the Begg test with STATA software and is presented in the form of funnel plots (Fig. 4B). The test results showed that *P = *.076, suggesting that there was no publication bias in any of the 9 studies.

### 3.2. Network pharmacology analysis

#### 3.2.1. Genes of EMs

According to the GeneCards database, a relevance score of ≥1.149, 1066 targets was confirmed. From the OMIM database, 432 targets were obtained. Duplicates were removed, and 1481 EMs targets were identified (Supplementary Material S4, Supplemental Digital Content, http://links.lww.com/MD/O19).

#### 3.2.2. Active components and targets of GZFL

After removing duplicates, 136 effective components of GZFL were collected from the TCMSP and BAT-Man databases. Among them, 23 kinds of *C cassia* Presl, and 33 kinds of *P cocos* (Schw.) Wolf, 19 kinds of *P suffruticosa* Andr., 40 kinds of *P lactiflora* Pall., and 21 kinds of *P persica* (L.) Batsch (Supplementary Material S5, Supplemental Digital Content, http://links.lww.com/MD/O20) were used. Chemical information and canonical SMILES of the components were collected and reviewed from the PubChem database. By searching the Swiss Target Prediction Database, 209 targets were identified. Combined with the 824 targets acquired from the BAT-Man database, 972 targets of GZFL were obtained after deduplication (Supplementary Material S5, Supplemental Digital Content, http://links.lww.com/MD/O20).

#### 3.2.3. Establishment of the biological components and target network of GZFL

**Figure 5.**
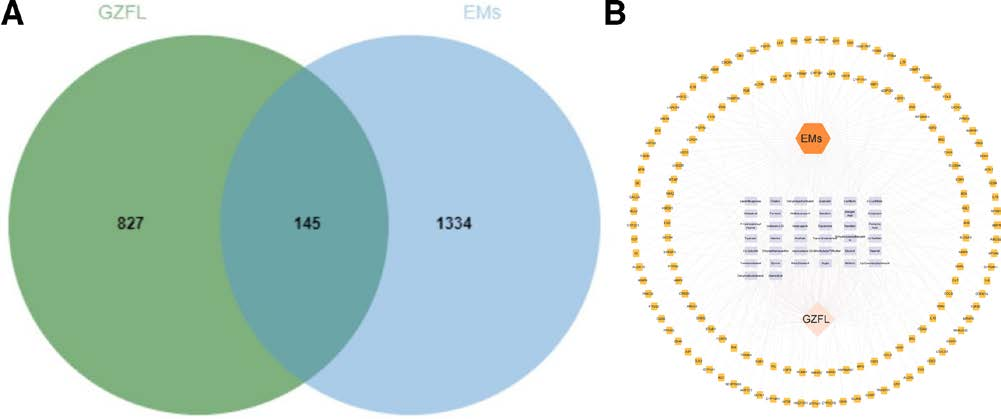
(A) The Venn diagram of targets of GZFL in TCMSP and BAT-man database and EMs targets from GeneCards and OMIM datasets. (B) The active ingredient-target network for 39 candidate bioactive ingredients and 145 potential targets of GZFL in EMs. EMs = endometriosis, GZFL = Guizhi Fuling capsule/pills, TCMSP = Traditional Chinese Medicine Systems Pharmacology.

One hundred forty-five targets of GZFL in the treatment of EMs were identified, as shown in a Venn diagram (Fig. 5A). Finally, 39 potential bioactive components were obtained, and the ingredient-target network, including 185 nodes and 435 edges, was constructed by Cytoscape (Fig. 5B). In the network, the yellow nodes represent the targets, while the purple nodes represent the corresponding biological components of the targets in GZFL.

#### 3.2.4. PPI network of GZFL and EMs common targets

**Figure 6.**
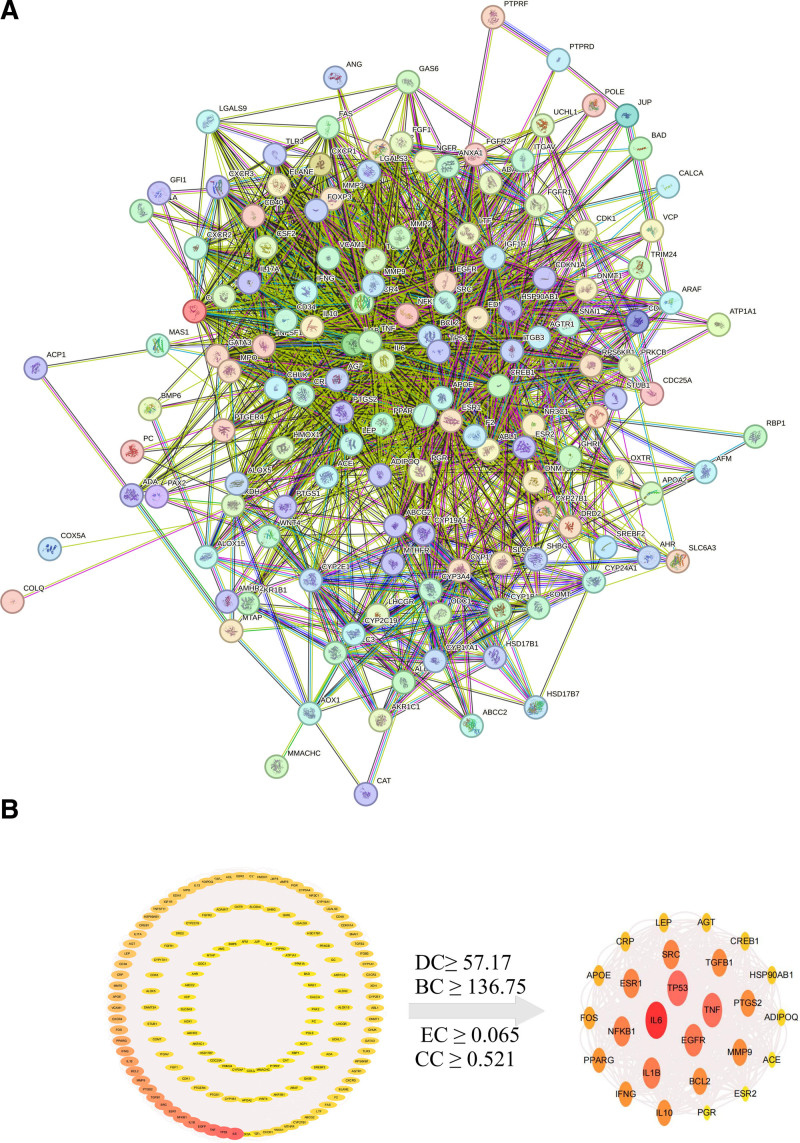
(A) A PPI network of 145 genes for GZFL treatment of EMs; (B) the top 26 key targets of the PPI network based on CytoNCA. EMs = endometriosis, GZFL = Guizhi Fuling capsule/pills, PPI = protein–protein interaction.

The 145 genes were uploaded to the STRING database, and the PPI network was constructed to represent the GZFL targets’ interactions when treated with EMs via Cytoscape (Fig. 6A,B). According to the CytoNCA algorithm, targets with DC, betweenness centrality, CC, and eigenvector centrality values higher than the average were obtained. Finally, we screened the top 26 genes with DC values as key targets, including interleukin-6 (IL-6), TP53, epidermal growth factor receptor (EGFR), Estrogen Receptor 1 (ESR1) or (ERα), Prostaglandin-endoperoxide synthase 2 (PTGS2) or (COX-2), MMP9, and ESR2, as shown in Figure 6C.

#### 3.2.5. GO functional annotation and KEGG pathway enrichment analysis

**Figure 7.**
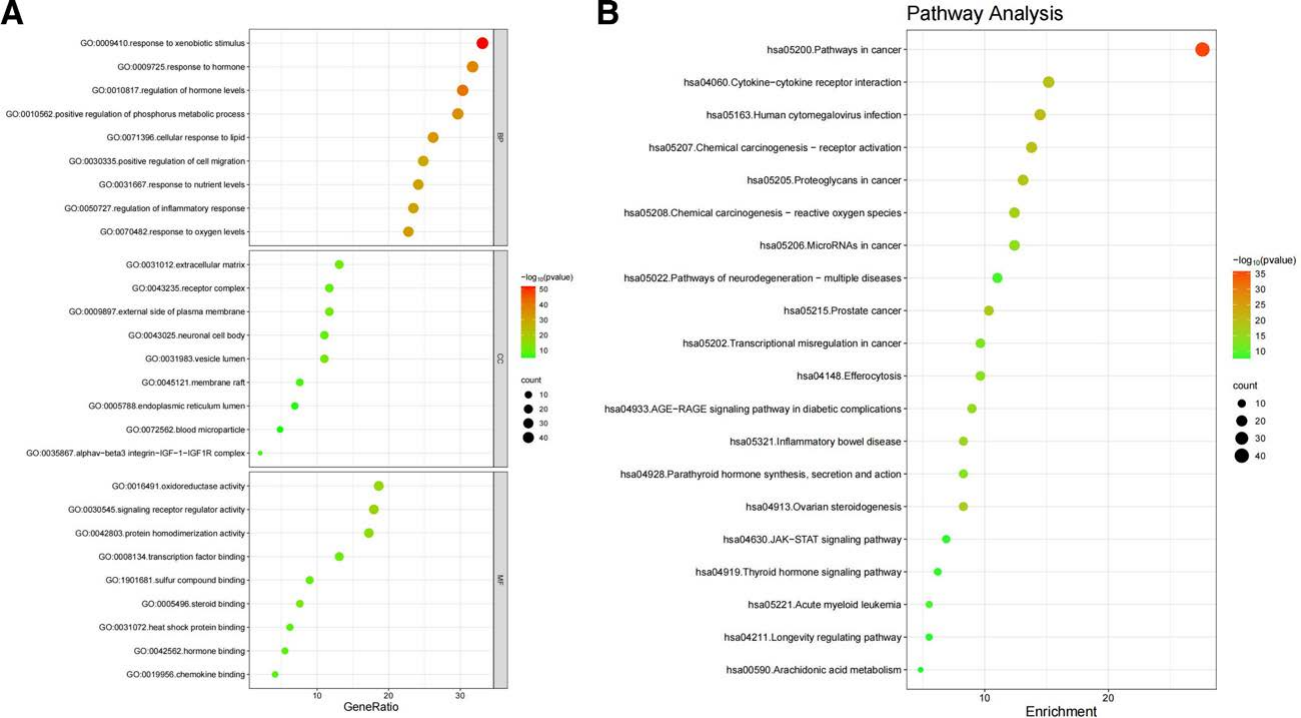
(A) GO analysis of 145 targets for GZFL treatment of EMs. (B) KEGG pathway enrichment analysis. EMs = endometriosis, GO = gene ontology, GZFL = Guizhi Fuling capsule/pills, KEGG = Kyoto Encyclopedia of Genes and Genomes.

The BP, CC, MF, and pathway terms for all 145 genes associated with GZFL treatment of EMs were obtained from Metascape. The number of cases for BP, MF, and CC were 693, 246, and 197, respectively (*P* < .01). According to the smallest *P*-value, the top ten biological annotations in GO were selected as prominent, with BPs focused on response to xenobiotic stimulus and hormone, regulation of hormone levels, and inflammatory response, while cellular components were mainly distributed in the receptor complex, and neuronal cell body, etc. Regarding MF, oxidoreductase activity, signaling receptor regulator activity, and transcription factor binding need to be noted (Fig. 7A). According to the KEGG pathway analysis, 308 signaling pathways (*P* < .01) were identified. The top 5 pathways were pathways in cancer, cytokine–cytokine receptor interaction, human cytomegalovirus infection, chemical carcinogenesis receptor activation, and proteoglycans in cancer, as shown in Figure 7B.

#### 3.2.6. Molecular docking verification

**Figure 8.**
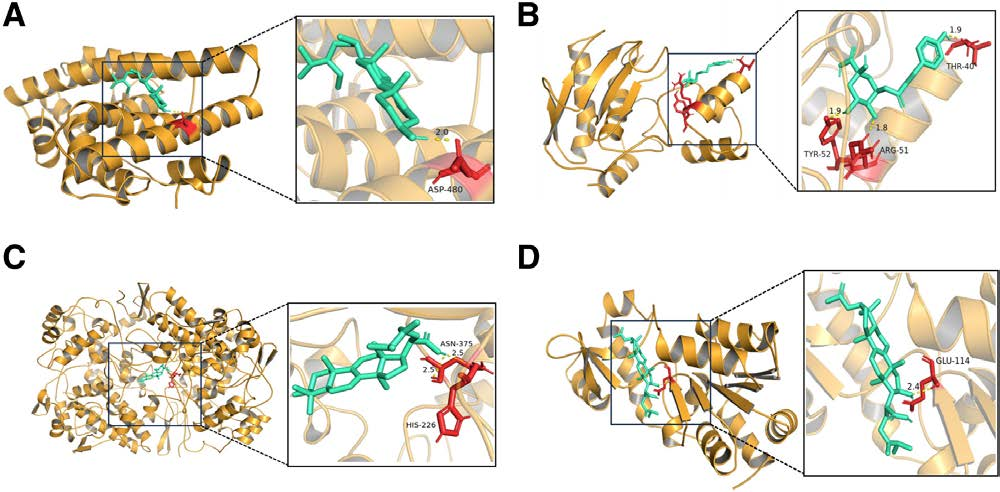
Pattern diagram of molecular docking. (A) Molecular docking of ESR1 and beta-sitosterol; (B) molecular docking of MMP9 and 3-p-Coumaroylquinic acid; (C) molecular docking of PTGS2 and dehydroeburicoic acid; (D) molecular docking of TP53 and pachymic acid. ESR1 = estrogen receptor alpha, MMP9 = matrix metalloproteinases-9, PTGS2 = cyclooxygenase-2, TP53 = cellular tumor antigen p53.

Four recognized GZFL target genes (TP53, ESR1, PTGS2, and MMP9) were identified based on the literature and PPI interaction network. We used AutoDock Vina to predict protein binding pockets and affinity for the best binding site (Table 2). The table shows that the binding energies of the 2 active ingredients were less than −4.25 kcal/mol, indicating good affinity for the key targets. Additionally, the binding energy of the 2 active ingredients were found to be less than −7 kcal/mol, indicating a strong docking affinity with the key targets. Furthermore, we plotted the docking patterns of these 4 “target protein-bioactive components” (Fig. 8). The molecular docking method further confirmed that the chemical components of pachymic acid, beta-sitosterol, dehydroeburicoic acid, and 3-p-coumaroylquinic acid in GZFL could be treated or improved in EMs by TP53, ESR1, PTGS2, and MMP9.

**Table 2:** Results of molecular docking.

Compound	Target gene	PDB ID	Protein pocket coordinates	Grid box size	Protein affinity (kcal/mol)
Pachymic acid	TP53	2H2D	X = −0.106,Y = 18.99,Z = 10.389	X = 44,Y = 60,Z = 42	−6.5
Beta-Sitosterol	ESR1	1X7R	X = 22.367,Y = 27.669,Z = 13.248	X = 48,Y = 46,Z = 58	−8
Dehydroeburicoicacid	PTGS2	3HS5	X = 26.698,Y = 27.878,Z = 46.98	X = 80,Y = 76,Z = 104	−9.4
3-p-Coumaroylquinic acid	MMP9	5TH6	X = −0.347,Y = 36.906,Z = 3.481	X = 44,Y = 42,Z = 50	−6.6

## 4. Discussion

EMs is a chronic systemic disease characterized by multiple clinical symptoms, including pelvic pain, dysmenorrhea, non-menstrual pelvic pain, and infertility.^[29–32]^ As the 2 classic symptoms, persistent pelvic pain affects 40% to 50% of women and adolescents,^[33]^ and 30% of patients with EMs have difficulty conceiving.^[34]^ In the present study, a meta-analysis of RCTs reported that GZFL combined with DNG was more clinically effective than DNG alone in the therapy of EMs, and network pharmacology analysis revealed that this effect may be related to hormone receptor regulation.

Estrogen is a key biological driver.^[35]^ Mediated by estrogen receptor (ER)β, estradiol allows endometrial tissue to attach to the peritoneum and promotes the generation of inflammatory mediators such as metalloproteinases, cytokines, or prostaglandins, as well as angiogenesis.^[36–38]^ Furthermore, progesterone resistance due to progesterone receptor deficiency in endometriotic stromal cells also plays an important role in EMs.^[39]^ Because of the high risk of disease recurrence and ovarian carcinogenesis, EMs require lifelong management^.[40]^ Clinical statistics indicate that 20% to 50% of women experience recurrence 5 years after surgery or medical treatment,^[41]^ while long-standing EMs is linked to a progressively elevated risk of ovarian cancer in women.^[42]^ Notably, during disease development, oxidative stress and hormone-dependent pathways in endometrioid histotypes seem to be 2 essential factors. Based on the above findings, the utilization of CA125 as a tumor marker may be consistent with the presence of recurring EMs, along with the level of E2 and MMP in patient serum, which may be important biomarkers of EMs. By overcoming progesterone resistance, progestin-based therapies are effective in the clinical treatment of EMs-associated pain.^[4]^

In traditional Chinese medicine, EMs include “*Zheng Jia* (gynecological mass in the abdomen),” “dysmenorrhea,” and “infertility.” Blood stasis is generally considered a crucial pathological factor, and activating blood and resolving stasis are regarded as the principles of treatment. GZFL, a classic prescription for promoting blood circulation, contributes to alterations in the metabolic profiles of the EMs rat model^[43]^ and alleviates menstrual pain severity in patients with primary dysmenorrhea.^[11]^ GZFL is not only an active compound with pharmacological effects in the treatment of EMs^[44]^ but also a Chinese herbal medicine and is recommended for nonpharmacological management.^[45]^

This meta-analysis provides a systematic review of studies on improving the clinical efficacy of GZFL therapy in patients with EMs. Specifically, GZFL combination therapy can reduce pain scores and cyst diameter in patients with EMs, simultaneously decreasing the levels of E2, CA125, and MMP in the serum. Notably, the studies included in this meta-analysis varied in dosage, patient age, and disease course; however, further subgroup analyses were not carried out due to the statistical insignificance of the meta-regression results, which we believe were fundamentally attributable to the limited sample sizes. Despite searching both Chinese and English databases, all the trials incorporated into our study were conducted in China, potentially introducing a selection bias. However, no publication bias was detected in any meta-analysis.

Network pharmacological studies revealed the relevant targets of GZFL136 potential bioactive components for the treatment of EMs, including IL-6, TP53, EGFR, ESR1 (ERα), PTGS2 (COX-2), and MMP9, and the potential mechanisms of action of these targets. EMs is an estrogen-dependent chronic inflammatory disorder.^[42]^ E2, produced by the ovary and endometriotic lesions, stimulates prostaglandins and perpetuates inflammation mediated by the ER.^[4]^ ESR1 transcript levels are significantly increased in all infertile women with EMs^[46]^; correspondingly, ectopic lesions in humans and mice can be suppressed by inhibiting ERβ.^[47]^ On the other hand, inflammation resulting from EMs caused by the perturbation of pro-inflammatory transcription factors, such as the chaperone protein FKBP4 or the coregulator Hic-5,^[48]^ could alter the progesterone signaling pathway and induce progesterone resistance. Additionally, steroid perturbation promotes endometriotic lesion formation by activating MMPs and angiogenesis.^[1]^ Therefore, regulating the levels of estrogen and progesterone and their receptors, reducing the secretion of COX-2, and further reducing prostaglandin production are therapeutic strategies for reducing pain in patients with EMs.

Ectopic lesions are also linked to excessive production of cytokines and chemokines. IL-6 treatment increases the number of endometriotic lesions by inducing NOTCH1 expression in ectopic glandular epithelial cells.^[49]^ In addition, therapies to downregulate pro-inflammatory cytokines (IL-1β, IL-6, and TNF-α) and oxidative stress markers can improve infertility in patients with EMs in clinical trials.^[50]^ Meanwhile, studies have identified that EGFR can promote the epithelial-mesenchymal transition by modulating the MMP, and inhibiting the expression of EGFR could significantly reduce the endometriotic lesions in a mouse model.^[51]^ In EMs-associated carcinogenesis, the levels of chemokines and cytokines may be potential driving factors. In ectopic tissues, gene mutations in overexpressed TP53 are also strongly associated with ovarian cancer.^[52,53]^

In conclusion, the potential bioactive components of GZFL regulate hormone levels and the inflammatory response via the cytokine–cytokine receptor interaction pathway as well as the cancer pathway through the above pathways, as shown by GO and KEGG analyses. Molecular docking of the key targets further validated possible treatment points. However, further animal experiments to verify the underlying mechanism are lacking in this study, and mass spectrometry analysis of GZFL components is also necessary, which needs to be further improved in the future.

In summary, this study demonstrated that GZFL combined with DNG is more clinically effective in the treatment of EMs than DNG alone, and the potential theoretical mechanism by which it functions may involve modulation of hormone receptors and inflammation. This study also provides new evidence for the utilization of GZFL in the management of EMs and offers a valuable reference for the clinical application of this formulation.

## 5. Conclusion

In this study, meta-analysis and network pharmacology technology were used to comprehensively assess the therapeutic effectiveness and potential mechanisms of GZFL on EMs. Meta-analysis indicated that GZFL combination therapy might improve the CER for the EMs treatment compared to DNG alone therapy. Specifically, GZFL reduces serum CA125, E2, and MMP in EMs patients, and reduces pain scores as well as the diameter of the ectopic cyst. Through network pharmacological analysis, we identified a total of 972 potential targets, 145 common targets, and 308 related pathways. Therefore, it can be inferred that GZFL is clinically significant for the treatment of EMs, as it might act on multiple targets and pathways. Further in vitro and in vivo studies are needed to confirm the biological mechanism of GZFL.

## Author contributions

**Data curation:** Yajie Qin, Xiaotian Yang.

**Investigation:** Xingran Tang.

**Methodology:** Huijin Zhao, Yang Jiao.

**Supervision:** Huifang Zhou.

**Visualization:** Qi Zhao.

**Writing – original draft:** Yajie Qin, Xiaotian Yang.

**Writing – review & editing:** Yajie Qin, Qi Zhao.

## Supplementary Material


