# Supplementary material for: Meta-analysis and network pharmacology studies of the clinical efficacy of Guizhi Fuling capsules/pills combined with dienogest in treating endometriosis
Source: Medicine (Baltimore). 2024 Dec 6;103(49):e40528. doi: 10.1097/MD.0000000000040528 (PMC11630926; doi:10.1097/MD.0000000000040528)
Supplement: Supplementary file 1 [file medi-103-e40528-s001.docx]

# S1 Search strategy

## Pubmed database

((("endometriosis"[MeSH Terms] OR "Endometrioses"[Title/Abstract] OR "Endometrioma"[Title/Abstract] OR "Endometriomas"[Title/Abstract] OR "endometriosis"[MeSH Terms]) AND "guizhi fuling capsule"[Title/Abstract]) OR "guizhi fuling capsule"[Text Word] OR "guizhi fuling decoction"[Title/Abstract] OR "guizhi fuling decoction"[Text Word] OR "guizhi fuling wan"[Title/Abstract] OR "guizhi fuling wan"[Text Word]) AND ("Dienogest"[Title/Abstract] OR "Dienogest"[Text Word])

## Embase database

1. endometriosis/exp OR endometriosis

2. 'chocolate cyst'/exp OR 'chocolate cyst' OR 'chocolate cyst of ovary'/exp OR 'chocolate cyst of ovary'

3. ('guizhi fuling capsule'/exp OR 'guizhi fuling capsule') OR ('guizhi fuling decoction'/exp OR 'guizhi fuling decoction')

4. 1 OR 2

5.4 AND 3

6.Dienogest

7.6 AND 5

## Cochrane Library

#1mesh descriptor endometriosis

#2(Endometrioses):ti,ab,kw OR (Endometrioma):ti,ab,kw OR (Endometriomas):ti,ab,kw OR (chocolate cyst of ovary):ti,ab,kw

#3 #1 OR #2

#4 (guizhi fuling capsule):ti,ab,kw OR (guizhi fuling capsule)):ti,ab,kw OR (guizhi fuling decoction):ti,ab,kw

#5 #3 AND #4

## Web Of Science database

((TS=("endometriosis" OR "chocolate cyst of ovary"OR "Endometrioses " OR "Endometrioma"OR" Endometriomas" OR "chocolate cyst")) AND TS=("guizhi fuling capsule" OR "guizhi fuling decoction"OR"guizhi fuling wan")) AND TS=("Dienogest")

## China National Knowledge Infrastructure(CNKI)


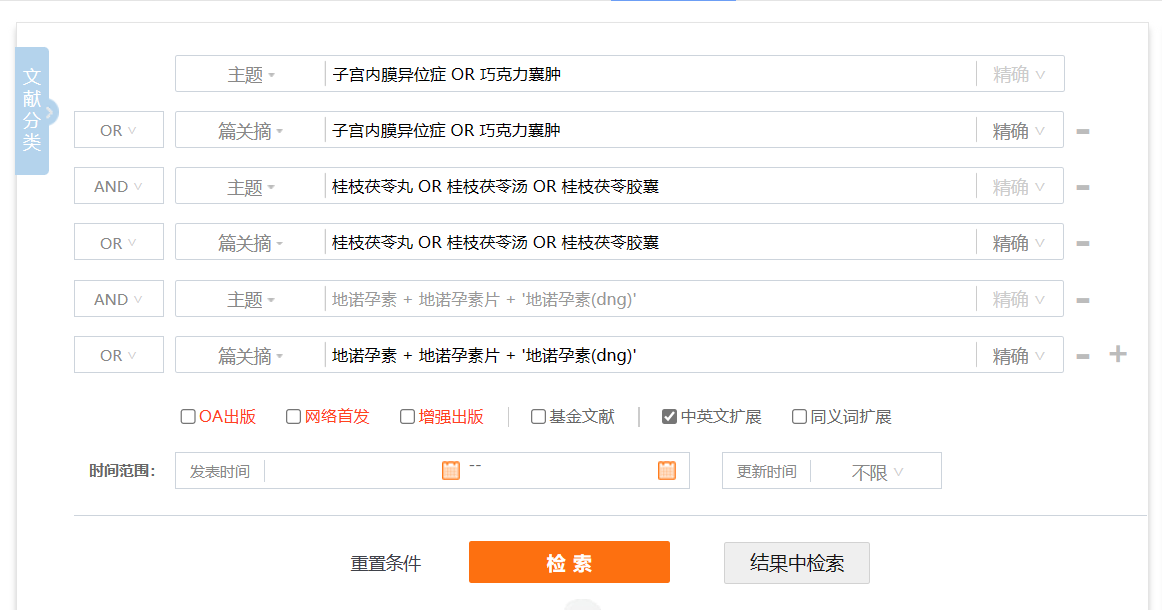


## Wanfang database

(主题:(子宫内膜异位症) or 题名或关键词:(子宫内膜异位 or 子宫内膜异位症 or 异位症 or 巧克力囊肿 or 巧囊) ) and (主题:(桂枝茯苓) or 题名或关键词:(桂枝茯苓丸 or 桂枝茯苓汤 or 桂枝茯苓胶囊 or 地诺孕素))

## Chinese Scientific Journals Database (VIP)

[((((((((题名或关键词=子宫内膜异位症 OR 题名或关键词=子宫内膜异位) OR 题名或关键词=异位症) OR 题名或关键词=巧克力囊肿) OR 题名或关键词=巧囊) AND (((题名或关键词=桂枝茯苓 OR 题名或关键词=桂枝茯苓丸) OR 题名或关键词=桂枝茯苓汤) OR 题名或关键词=桂枝茯苓胶囊)) AND 题名或关键词=地诺孕素) OR ((((摘要=子宫内膜异位症 OR 摘要=子宫内膜异位) OR 摘要=异位症) OR 摘要=巧克力囊肿) OR 摘要=巧囊)) AND (((摘要=桂枝茯苓 OR 摘要=桂枝茯苓丸) OR 摘要=桂枝茯苓汤) OR (摘要=桂枝茯苓胶囊 AND 摘要=地诺孕素)))](https://qikan.cqvip.com/Qikan/search/index?LngMySearHistoryIdGuid=689846c4-3070-4fc6-9ba1-dc418584b04e&from=Qikan_Article_History)
