# Supplementary material for: Meta-analysis and network pharmacology studies of the clinical efficacy of Guizhi Fuling capsules/pills combined with dienogest in treating endometriosis
Source: Medicine (Baltimore). 2024 Dec 6;103(49):e40528. doi: 10.1097/MD.0000000000040528 (PMC11630926; doi:10.1097/MD.0000000000040528)
Supplement: Supplementary file 2 [file medi-103-e40528-s002.docx]

# Table.1 Meta-regression analysis of the serum CA125 based on age and disease duration(Performed by STATA software)

| _ES | exp(b) | Std. Err. | t | P>\|t\| | [95% Conf. Interval] | |
| --- | --- | --- | --- | --- | --- | --- |
| Age | .6896555 | .3556711 | -0.72 | 0.523 | .1336109 | 3.559774 |
| _cons | .3183862 | .2371197 | -1.54 | 0.222 | .0297587 | 3.406388 |

| _ES | exp(b) | Std. Err. | t | P>\|t\| | [95% Conf. Interval] | |
| --- | --- | --- | --- | --- | --- | --- |
| Disease duration | 1.230905 | .647101 | 0.40 | 0.719 | .2310084 | 6.558753 |
| _cons | .1383964 | .1198297 | -2.28 | 0.107 | .0087988 | 2.176829 |

# **Table.2 Meta-regression analysis of the Diameter of the ectopic cyst based on disease course or dosage of GZFL(Performed by STATA software)**

| _ES | exp(b) | Std. Err. | t | P>\|t\| | [95% Conf. Interval] | |
| --- | --- | --- | --- | --- | --- | --- |
| Disease duration/ Dosage | 2.924047 | .7915118 | 3.96 | 0.157 | .0938103 | 91.14189 |
| _cons | .044286 | .0167869 | -8.22 | 0.077 | .0003585 | 5.470026 |
