# Supplementary material for: Meta-analysis and network pharmacology studies of the clinical efficacy of Guizhi Fuling capsules/pills combined with dienogest in treating endometriosis
Source: Medicine (Baltimore). 2024 Dec 6;103(49):e40528. doi: 10.1097/MD.0000000000040528 (PMC11630926; doi:10.1097/MD.0000000000040528)

# S2. Sensitivity analyses

## Clinical efficiency rate


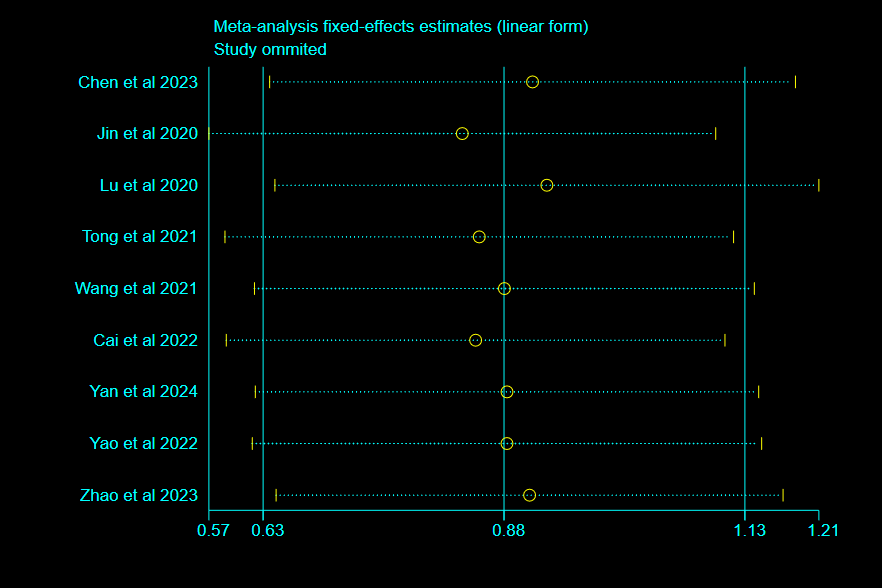


## CA125
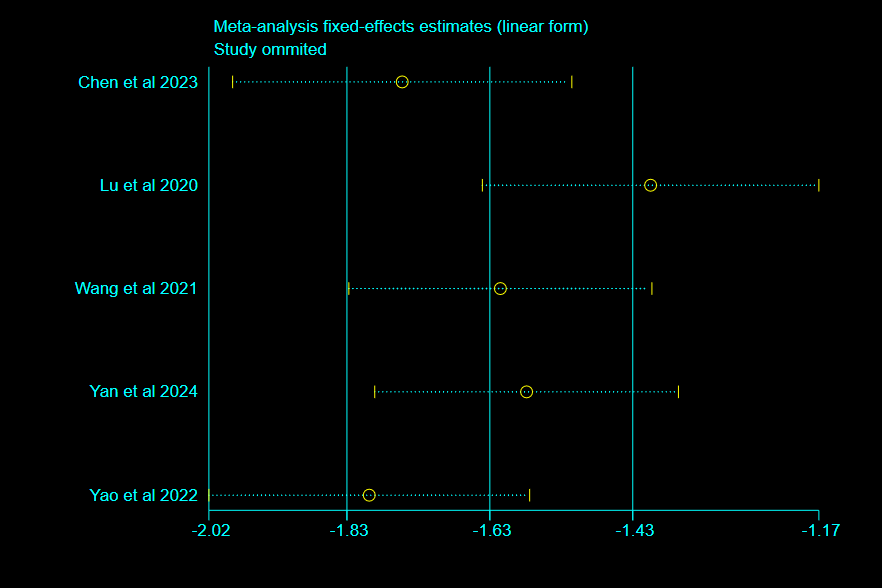


## E2

Before removing Chen et al.'s research


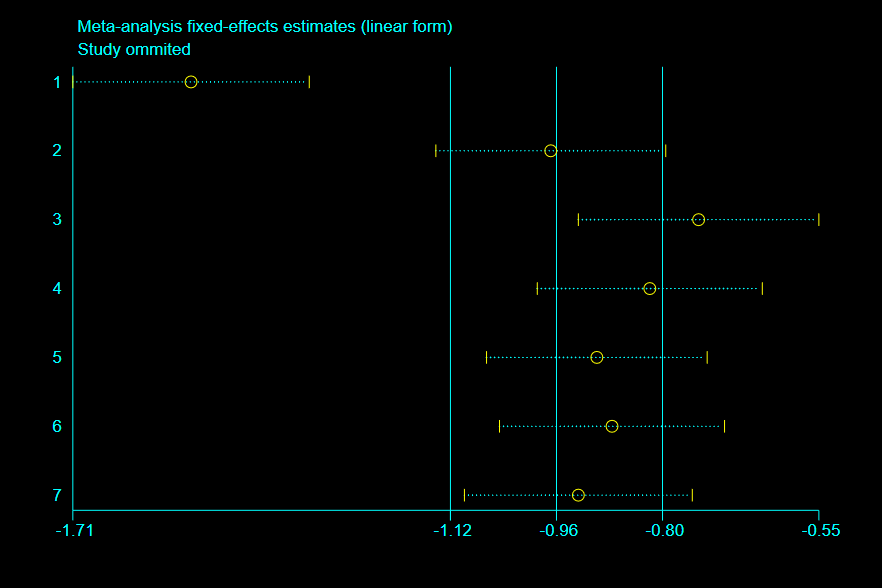


After removing Chen et al.'s research


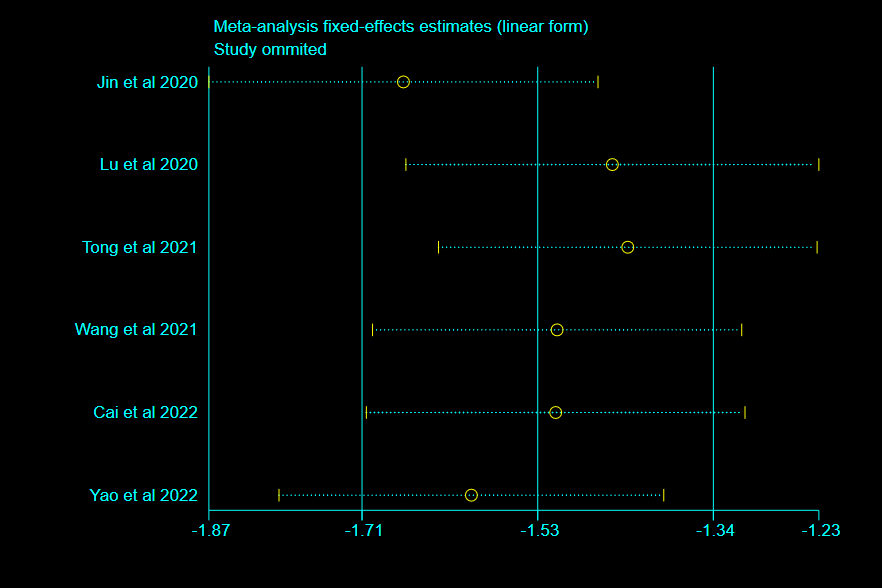


## Pain scores


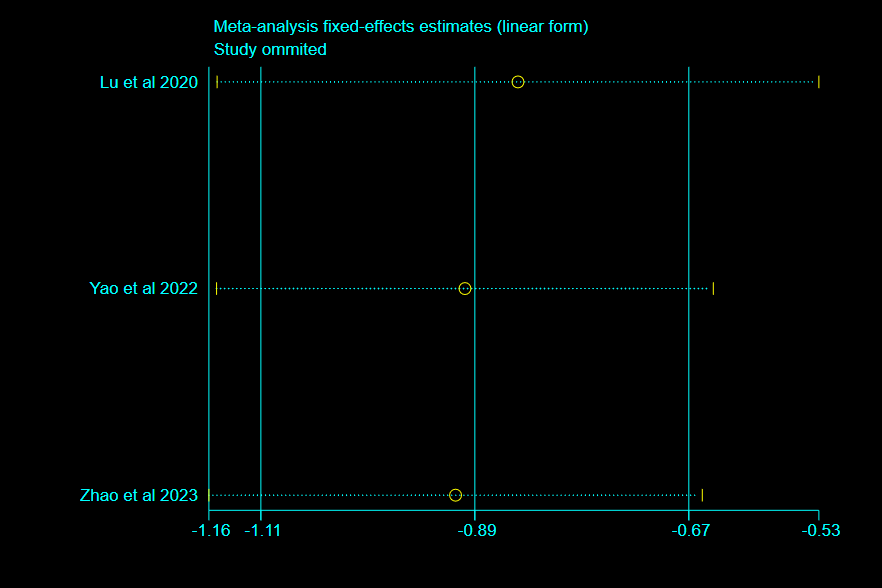


## MMP


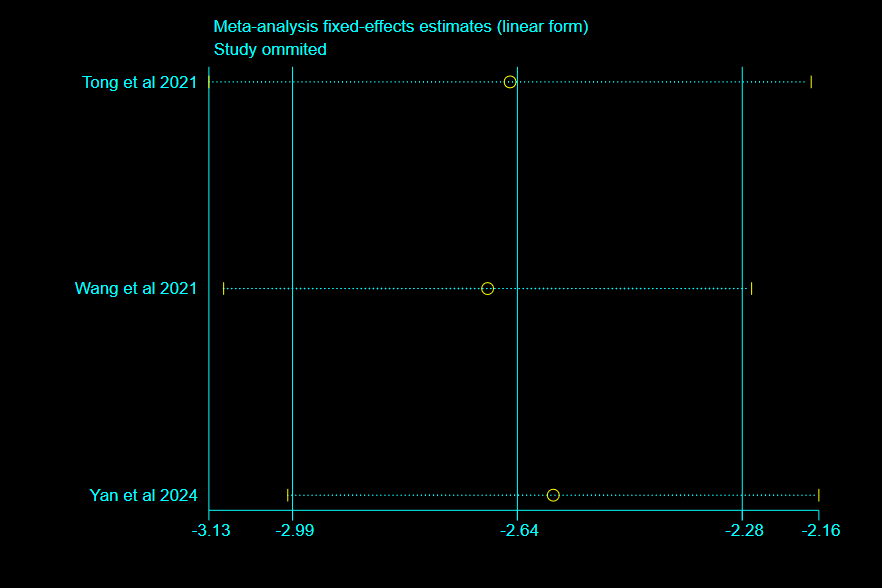


## Diameter of the ectopic cyst


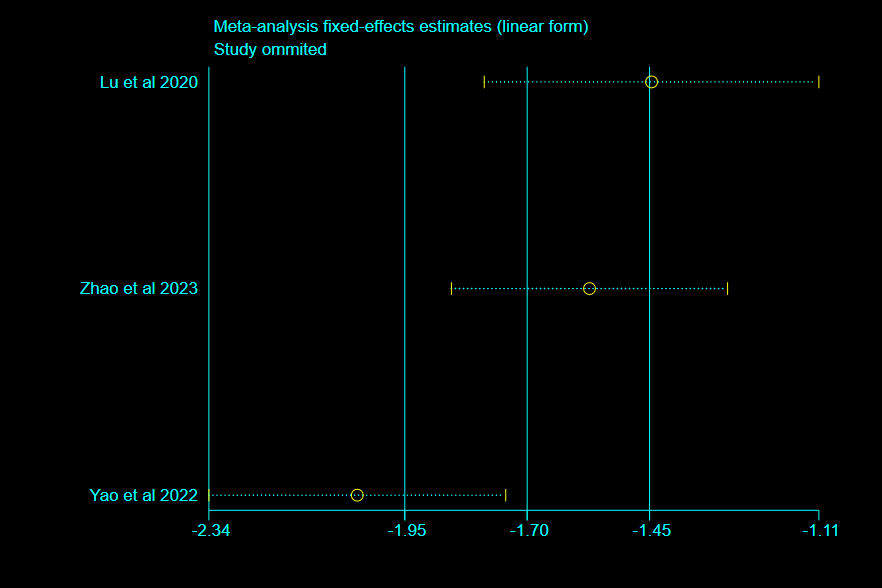

Supplement: Supplementary file 3 [file medi-103-e40528-s003.docx]
